# Circulating Lipoproteins Are a Crucial Component of Host Defense against Invasive *Salmonella typhimurium* Infection

**DOI:** 10.1371/journal.pone.0004237

**Published:** 2009-01-21

**Authors:** Mihai G. Netea, Leo A. B. Joosten, Monique Keuter, Frank Wagener, Anton F. H. Stalenhoef, Jos W. M. van der Meer, Bart Jan Kullberg

**Affiliations:** 1 Division of General Internal Medicine, Department of Medicine, Radboud University Nijmegen Medical Center, Nijmegen, The Netherlands; 2 Nijmegen Institute of Infection, Inflammation and Immunity (N4i), Nijmegen, The Netherlands; 3 Department of Pharmacology and Toxicology, Nijmegen Center for Molecular Life Sciences, Radboud University Nijmegen Medical Center, Nijmegen, The Netherlands; University of California Merced, United States of America

## Abstract

**Background:**

Circulating lipoproteins improve the outcome of severe Gram-negative infections through neutralizing lipopolysaccharides (LPS), thus inhibiting the release of proinflammatory cytokines.

**Methods/Principal Findings:**

Low density lipoprotein receptor deficient (LDLR−/−) mice, with a 7-fold increase in LDL, are resistant against infection with *Salmonella typhimurium* (survival 100% vs 5%, *p*<0.001), and 100 to 1000-fold lower bacterial burden in the organs, compared with LDLR+/+ mice. Protection was not due to differences in cytokine production, phagocytosis, and killing of *Salmonella* organisms. The differences were caused by the excess of lipoproteins, as hyperlipoproteinemic ApoE−/− mice were also highly resistant to *Salmonella* infection. Lipoproteins protect against infection by interfering with the binding of *Salmonella* to host cells, and preventing organ invasion. This leads to an altered biodistribution of the microorganisms during the first hours of infection: after intravenous injection of *Salmonella* into LDLR+/+ mice, the bacteria invaded the liver and spleen within 30 minutes of infection. In contrast, in LDLR−/− mice, *Salmonella* remained constrained to the circulation from where they were efficiently cleared, with decreased organ invasion.

**Conclusions:**

plasma lipoproteins are a potent host defense mechanism against invasive *Salmonella* infection, by blocking adhesion of *Salmonella* to the host cells and subsequent tissue invasion.

## Introduction

*Salmonella* infections are a significant cause of morbidity and mortality, despite preventive measures and the availability of antibiotics. A major virulence factor of *Salmonella* is lipopolysacharide (LPS) [1], [2], and *Salmonella* strains with a reduced LPS expression have a poor growth under stress conditions and are less virulent [2]. In addition, LPS induces proinflammatory cytokines and is essential for internalization of *Salmonella* by host cells [3]. Interaction of LPS with cellular receptors is essential and therefore, strategies aimed at blocking this interaction may have a therapeutic potential in invasive infections.

Lipoproteins bind and neutralize bacterial LPS, and they prevent the induction of potentially harmful proinflammatory cytokines such as IL-1β and TNFα [4]. In experimental models, administration of lipoproteins protects against endotoxic shock [5]–[7]. Low density lipoprotein receptor deficient (LDLR−/−) mice have a 7 times higher LDL-cholesterol level than control mice. We have shown previously that LDLR−/− mice survive longer and have lower proinflammatory cytokine concentrations than control mice after LPS challenge, as well as after infection with *Klebsiella pneumoniae* [8]. In addition, LDL administration can protect against Gram-negative microorganisms through its neutralizing effects on LPS [9].

Although *Salmonella* is a Gram-negative organism, the clinical picture and inflammatory response in systemic *Salmonella* infections (e.g., typhoid fever) differs from that in other Gram-negative sepsis [10]. This is most likely due to the behaviour of *Salmonella* as facultative intracellular pathogens, and to the fact that the pattern of cytokine induction differs from other Gram-negative infections. In mice, TNFα is undetectable in the circulation until several days after *S. typhimurium* infection, whereas TNFα rises at 1 hour after extracellular Gram-negative infection [11]. The level of cytokinemia during *Salmonella* infections does not reach the toxic levels seen in endotoxic shock, and inhibition of TNFα during *Salmonella* infection worsens the outcome [12], [13].

Considering these differences in the pathogenesis of *Salmonella* and extracellular Gram-negative infections, one would envisage either beneficial effects of lipoproteins on the host resistance to *Salmonella* through blockade of cellular internalization, or deleterious effects by blocking the induction of cytokines by *Salmonella* LPS that are required for the activation of host defense. In the present study, we investigated the effect of lipoproteins on the outcome of *Salmonella* infection.

## Methods

### Animals

Homozygous C57Bl/6J mice lacking low density lipoprotein receptors (LDLR−/−) and their wild-type littermates (C57Bl/6J LDLR+/+) were obtained from Jackson Laboratory (Bar Harbour, ME) [8]. Homozygous apolipoprotein E (ApoE)-deficient mice on a C57Bl/6 background were obtained from the Transgenic Facility of Leiden University Medical Center, Leiden, The Netherlands [14]. Six to eight weeks old littermate LDLR+/+ and LDLR−/− mice were used, weighing 20–25 grams. The animals were fed standard laboratory chow and housed under specific pathogen free conditions. The experiments were approved by the Ethics Committee for animal experiments at the Radboud University Nijmegen.

### *Salmonella typhimurium* infection

A serum-resistant strain of *S. typhimurium* (phage type 510) was grown by overnight incubation at 37°C in nutrient broth (BHI Oxoid). Mice were injected i.v. or i.p. with 1×10^2^ cfu of *S. typhimurium*. Survival was assessed daily for 21 days in groups of at least 20 animals. On day 1, 3 and 7 after infection, mice were killed by cervical dislocation and blood for cytokines or organs for outgrowth of the microorganisms were collected. For this purpose, the liver and spleen were removed aseptically, and bone marrow was flushed from the femur aseptically with 1 ml of sterile saline. The number of viable *Salmonella* organisms was determined by plating several dilutions on Brilliant Green agar (BGA) plates. The results were expressed as log cfu per gram of tissue.

### Distribution of *Salmonella* cfu

In a separate experiment, *Salmonella* cfu (10^5^/mouse) were injected i.v. Distribution was determined in blood, liver and spleen after 30, 60, 120 and 360 minutes by plating serial dilutions of blood and homogenized tissue samples on BGA plates. Groups of 5 mice were used for each time point.

### Intracellular killing of *S. typhimurium* by peritoneal phagocytes

Phagocytosis and intracellular killing of *S. typhimurium* was assessed in vitro using peritoneal macrophages and PMN of LRLR+/+ and LDLR−/− mice. Exudate peritoneal neutrophils (PMN) were harvested 4 h after an i.p. injection of 10% proteose peptone, and exudate macrophages 72 h after i.p. injection of proteose peptone. 5×10^5^ cells in 100 µl of RPMI were dispensed into 96-well flat bottom plates (Costar) and incubated at 37°C and 5% CO_2_. To assess phagocytosis, 1×10^5^ *Salmonella* organisms/mL were incubated on the phagocyte monolayers at 37°C in RPMI with 10% serum. After 15 min, supernatants were aspirated and the monolayers were gently washed with medium to remove uningested bacteria. The supernatants were plated on BGA agar (the non-phagocytozed fraction). To assess intracellular killing, the wells containing the cells with phagocytosed bacteria were scraped with a plastic paddle and washed with 200 µl distilled H_2_O to lyse the phagocytes. The number of viable bacteria was determined by plating serial dilutions on BGA plates.

### In vitro cytokine production

Resident peritoneal macrophages were harvested from peritoneal cavity, and cells were resuspended in RPMI 1640 containing 1 mM pyruvate, 2 mM L-glutamine and 100 µg gentamicin per ml, and incubated (10^5^/well) in 96-wells microtiter plates (Costar). Heat-killed (30 min, 100°C) *S. typhimurium* (10^6^ microorganisms in 100 µL of RPMI) were added to peritoneal macrophages and incubated at 37°C in 5% CO2. After 24 h, the supernatants were collected and stored at −70°C until assayed. To the macrophages in the monolayer, 200 µL of RPMI was added and the cells were disrupted by three freeze-thaw cycles to determine the cell-associated cytokine contents.

### Cytokine measurements

TNFα, IL-1α and IL-1ß concentrations were determined using specific radioimmunoassays (RIA), as previously described [8]. To assess cytokine mRNA expression, total RNA from spleen cells 24 hours after infection was isolated as described [15]. The following primers were used for the PCR reactions: GAPDH, sense, 5′–AACTCCCTCAAGATTGTCAGCA–3′, and antisense, 5′–TCCACCACCCTGTTGCTGTA–3′; TNFα, sense, 5′–TCTCATCAGTTCTATGGCCC–3′, and antisense, 5′–GGGAGTAGACAAGGTACAAC– 3′; IL-1α, sense, 5′-CAGTTCTGCCATTGACCATC-3′, and antisense, 5′-TCTCACTGAAACTCAGCCGT-3′, IL–1β, sense, 5′–TTGACGGACCCCAAAAGATG–3′, and antisense, 5′–AGAAGGTGCTCATGTCCTCA–3′ (Eurogentec, Seraing, Belgium). After checking the reactions to be in the log phase, thirty PCR cycles were performed with sets at 92°C for 30 sec., 55°C for 30 sec., and 72°C for 90 sec., using a Mastercycler 5330 (Eppendorf). PCR products were run on 2% agars gels stained with ethidium bromide. The gels were scanned on a densitometer (GS–670, Bio-Rad) and analyzed using Molecular Analyst software (Bio-Rad). The relative amount of TNFα, IL-1α and IL–1β mRNA in a sample was expressed as a ratio versus the amount of mRNA for the housekeeping gene GAPDH.

### Growth of *Salmonella* in vitro

To investigate the effect of lipoproteins on microbial growth in vitro, 0.5×10^3^ cfu *S. typhimurium* in 0.5 mL BHI were incubated with 0.5 mL of plasma obtained from control C57Bl/6J mice, or from LDLR−/− mice and ApoE−/− mice. After 2, 7, 12 and 24 hours, aliquots of 0.1 mL were removed, serial dilutions were plated on BGA agar, and cfu were counted after overnight incubation at 37°C.

### Effect of lipoproteins on interaction of *Salmonella* with monocytes and endothelial cells

To assess the effect of lipoproteins on the production of cytokines, *S. typhimurium* LPS (10 ng/mL; Sigma) and heat-killed (30 min, 100°C) *S. typhimurium* (10^7^ organisms/mL) were preincubated with lipoprotein-depleted plasma (LPDP) or isolated LDL [16] at various concentrations for 60 min, before being added to the macrophages of LDLR+/+ mice (10^5^/well). The production of TNFα after 24 h stimulation was measured as described above, and expressed as relative TNF production compared to controls in LPDP.

To assess the effect of lipoproteins on the attachment of *Salmonella* to vascular endothelial cells, *S. typhimurium* were resuspended to 6×10^8^/ml in 0.01 mg/ml FITC (Fluka) in 0.05 M carbonate-bicarbonate buffer (pH 9.5). After incubation for 15 min at room temperature in the dark, FITC-labeled *Salmonella* cells were washed twice in PBS containing 1% BSA and subsequently incubated with isolated 1.1 mmol/L LDL for 4 hours, or with LPDP as a negative control. The human endothelial cell line (HMEC-1) (CDC Atlanta, GA) was cultured in MCDB131 medium supplemented with 10% fetal calf serum, EGF (10 ng/ml), hydrocortison (1 µg/ml), and glutamine at 37°C and 5% CO_2_,. 1×10^5^ HMEC-1 cells were trypsinized and incubated for 1 hour with 3×10^9^ FITC-labeled *S. typhimurium*, which were preincubated with either LDL or LPDP. After incubation, the non-bound *Salmonella* was thoroughly washed off, after which the cells were fixated with 2% paraformaldehyde in PBS and analyzed for binding of FITC-labeled *Salmonella* by flow cytometry using the FACScalibur (BD Biosciences).

### Statistical analysis

Survival of groups of mice was compared by the Kaplan-Meyer log-rank test. Differences in concentrations of cytokines and in organ counts of the microorganisms were analyzed by the Mann-Whitney U test. Differences were considered significant at *P*<.05. All the experiments were at least performed in duplicate.

## Results

### Outcome of *Salmonella* infection in LDLR−/− mice

**Figure 1:**
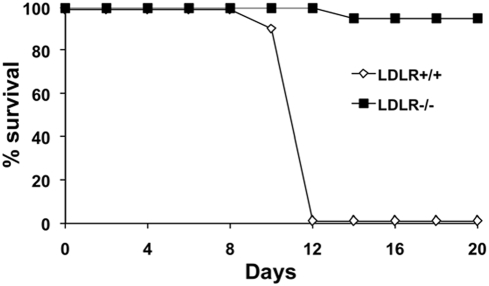
LDLR−/− mice are more resistant to *S. typhimurium* infection. Survival of LDLR−/− and LDLR+/+ C57Bl/6J mice after i.v. injection of 10^2^ *S. typhimurium*. n = 20/group.

**Figure 2:**
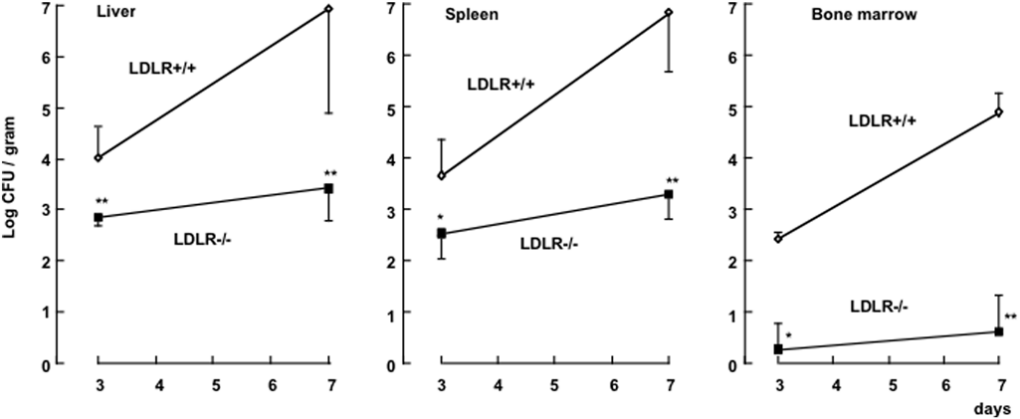
Outgrowth of *S. typhimurium* in the organs of LDLR+/+ and LDLR−/− mice. Outgrowth of *S. typhimurium* in the liver, spleen and bone marrow of LDLR−/− and control (LDLR+/+) C57Bl/6J mice after i.v. injection of 10^2^ cfu. Each point represents the mean±SD for at least 10 animals. Significant differences between LDLR−/− and LDLR+/+ mice are indicated (*, P<.01; **, *P*<.001; Mann-Whitney U test).

The total cholesterol concentrations were significantly higher in the uninfected LDLR−/− mice than in their wild-type littermates (9.6±1.1 mmol/L vs. 2.3±0.5 mmol/L). After i.v. infection with 10^2^ cfu of *S. typhimurium*, only 5% of the LDLR−/− mice died, whereas the mortality of control LDLR+/+ was 100% within 12 days of infection (*P*<.001; Fig. 1). A similar difference in mortality was apparent when mice were infected intraperitoneally with *S. typhimurium* (10% mortality in LDLR−/− mice, vs. 100% mortality in control LDLR+/+ mice, p<0.01). The reduced mortality to infection in LDLR−/− mice was accompanied by a markedly reduced bacterial load in the organs (Fig. 2). On day 7 of infection, the differences between the control and LDLR−/− mice approached 10,000-fold (*P*<.001; Fig. 2).

### Intracellular killing of *Salmonella* by cells from LDLR+/+ and LDLR−/− mice in vitro

**Figure 3:**
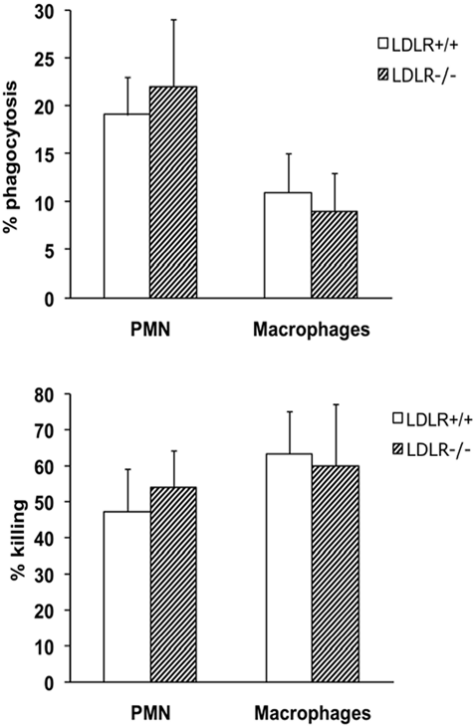
Phagocytosis and killing of *S. typhimurium* by neutrophils and macrophages of LDLR−/− mice. Phagocytosis of *S. typhimurium* after 15 min incubation, and intracellular killing of *S. typhimurium* after 4 h, by proteose peptone-elicited peritoneal neutrophils and macrophages. Data are expressed as percentage of the initial number of microorganisms. No significant differences between LDLR−/− and LDLR+/+ were found.

The numbers of *Salmonella* CFU phagocytized by neutrophils and macrophages of LDLR−/− and LDLR+/+ mice were similar (Fig. 3A). In addition, the intracellular killing assay demonstrated that neutrophils and macrophages of LDLR−/− mice and LDLR+/+ mice did not differ in their ability to kill *S. typhimurium* intracellularly (Fig. 3B). The killing rate did not differ when lipoprotein-rich serum of LDLR−/− mice was coincubated with LDLR+/+ control macrophages, and likewise, serum from LDLR+/+ mice did not affect the killing of *Salmonella* by LDLR−/− macrophages (not shown).

### Circulating cytokines during *Salmonella* infection

**Figure 4:**
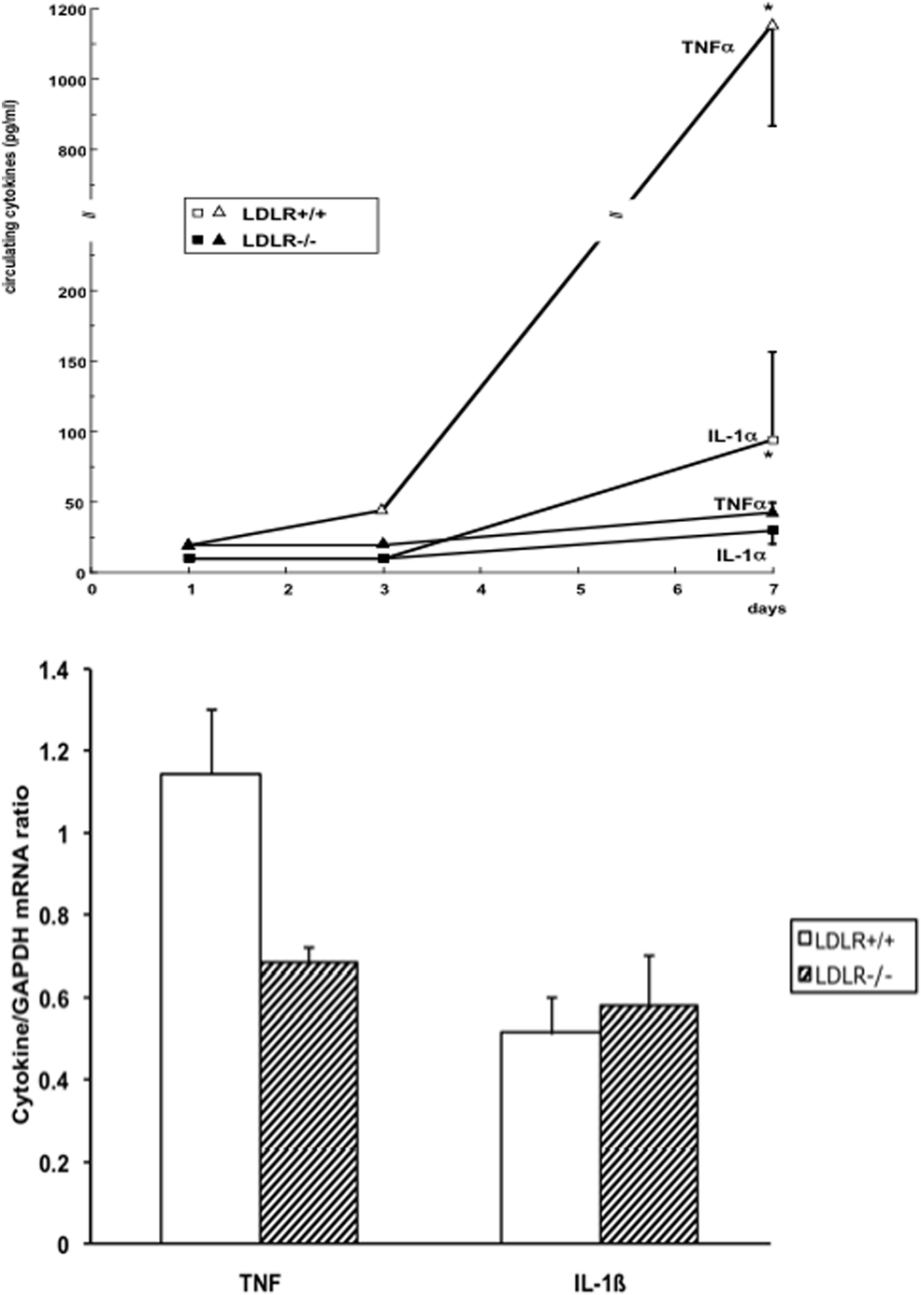
In-vivo cytokine production in LDLR+/+ and LDLR−/− mice infected with *S. typhimurium*. LDLR−/− and control (LDLR+/+) C57Bl/6J mice were infected i.v. with 10^2^ cfu of *S. typhimurium*. After 1, 3 or 7 days, groups of 5 mice/group were sacrificed, and circulating concentrations of cytokines in the plasma were measured by specific ELISA. In a separate experiment, TNF and IL-1β mRNA in the spleens of LDLR+/+ and LDLR−/− mice was assessed by semi-quantitative RT-PCR, and expressed as ratio to GAPDH mRNA expression. Data are presented as means±SD, *p<0.05.

On day 1, cytokine concentrations in all samples were under the detection limit. No detectable concentrations of IL-1ß (<20 pg/ml) were found at any time point during the infection. On day 3, IL-1α and TNFα were under the detection limit in LDLR−/− mice, while TNFα concentrations tended to be slightly higher (45±10 pg/ml) in LDLR+/+ mice (n.s.). On day 7, circulating concentrations of IL-1α and TNFα were significantly higher in LDLR+/+ than in LDLR−/− mice: 95±63 pg/ml vs 30±10 pg/ml for IL-1α (*P*<0.02) and 1140±290 pg/ml vs 43±6 pg/ml for TNFα (*P*<0.01) (Fig. 4A). These differences were most likely due to the greater amounts of *Salmonella* in the LDLR+/+ mice, leading to increased cytokine stimulation.

Importantly, the differences in cytokine concentrations were not secondary to an intrinsically deficient cytokine production in the organs of the LDLR−/− mice: the amounts of TNF and IL-1β mRNA in the spleens of LDLR+/+ and LDLR−/− mice on day 1 of infection was similar (Fig. 4B). No differences in the expression of IL-1α mRNA were observed either (not shown). In line with this, peritoneal macrophages of LDLR−/− and LDLR+/+ mice stimulated with heat-killed *Salmonella* produced similar amounts of TNFα in vitro: unstimulated macrophages, 194±91 pg/ml and 186±55 pg/ml, macrophages coincubated with heat-killed *Salmonella*, 550±166 pg/ml and 520±135 pg/ml for LDLR−/− and LDLR+/+ mice, respectively (*P*>.05).

### Resistance of ApoE−/− mice to *Salmonella* infection

**Figure 5:**
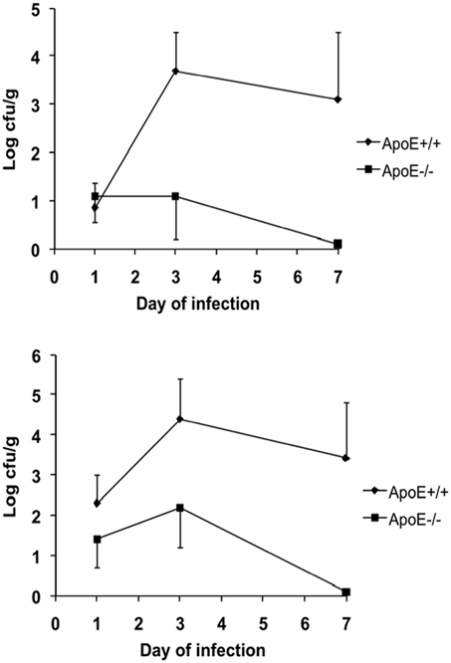
Outgrowth of *S. typhimurium* in the organs of ApoE+/+ and ApoE−/− mice. Outgrowth of *S. typhimurium* in the liver and spleen of ApoE−/− and control (ApoE+/+) C57Bl/6J mice after i.v. injection of 10^2^ cfu. Each point represents the mean±SD for at least 10 animals. Significant differences between ApoE−/− and ApoE+/+ mice were found for both liver and spleen on days 3 and 7 (p<0.01).

To investigate whether the increased lipoprotein concentrations in another model of hyperlipoproteinemia can also protect against salmonellosis, hyperlipoproteinemic ApoE−/− mice (total serum cholesterol, 16.1±3.7 vs. 1.9±0.2 mmol/L [14]) were infected i.v. with 10^2^ cfu of *S. typhimurium*. Whereas little differences were apparent on day 1 of infection, the outgrowth of *Salmonella* on day 3 and 7 after the infection was 100 to 1000-fold less in the liver and spleen of ApoE−/− mice compared to that in ApoE+/+ control animals (Fig. 5).

### Effect of lipoproteins on the growth of *S. typhimurium* in vitro

**Figure 6:**
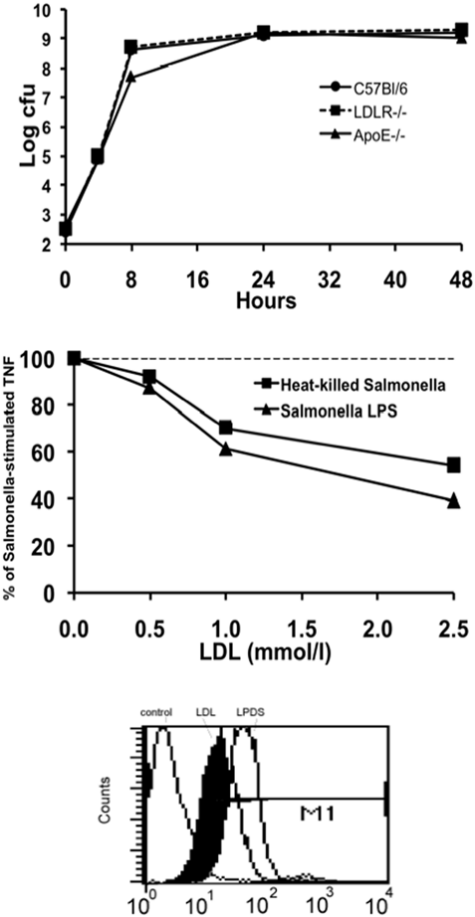
The effect of LDL on the interaction between *S. typhimurium* and the host cells. The growth of *S. typhimurium* was identical in plasma harvested from control C57Bl/6, LDLR−/− or ApoE−/− mice (upper panel). Preincubation of *Salmonella* LPS or heat-killed *S. typhimurium* with various concentrations of LDL led to a significantly diminished stimulation of TNF when added on normal macrophages (middle panel). Similarly, preincubation of FITC-labelled *S. typhimurium* with LDL led to a diminished adhesion of the microorganisms to vascular endothelial cells, when compared with the lipoprotein-deficient serum (LPDS).

To test whether lipoproteins have a direct inhibitory effect on the growth of *Salmonella*, plasma isolated from control mice (cholesterol concentration 2.3 mmol/L) and hyperlipoproteinemic plasma from either LDLR−/− or ApoE−/− mice (cholesterol concentrations, 9.6 and 16.1 mmol/L) was added to the culture. The growth curves of *Salmonella* were similar in broth with plasma obtained from all mouse strains (Fig. 6A).

### Inhibition of the interaction of *Salmonella* with monocytes and endothelial cells by lipoproteins

Because lipoproteins are known to bind and neutralize LPS, we preincubated *Salmonella* LPS and heat-killed whole *Salmonella* bacteria with LDL at various concentrations, and subsequently stimulated normal (LDLR+/+) macrophages for cytokine production. As shown in Fig. 6B, TNFα production induced by *S. typhimurium* is reduced in the presence of elevated LDL concentrations. To assess the effect of lipoproteins on the attachment of *Salmonella* to endothelial cells, FITC-labelled *S. typhimurium* was preincubated with LDL. Their attachment to endothelial cells was significantly reduced compared to that of *Salmonella* preincubated with lipoprotein-free plasma, as shown by both reduction of the percentage of cells binding *Salmonella* (97% cells bound LDPD-*Salmonella*, whereas only 82% cells bound LDL-*Salmonella*), as well as the mean fluorescence intensity per cell (41% reduction, from 56 to 33 conventional units) (see also Fig. 6C).

### Protection against organ invasion by *Salmonella* in LDLR−/− mice

**Figure 7:**
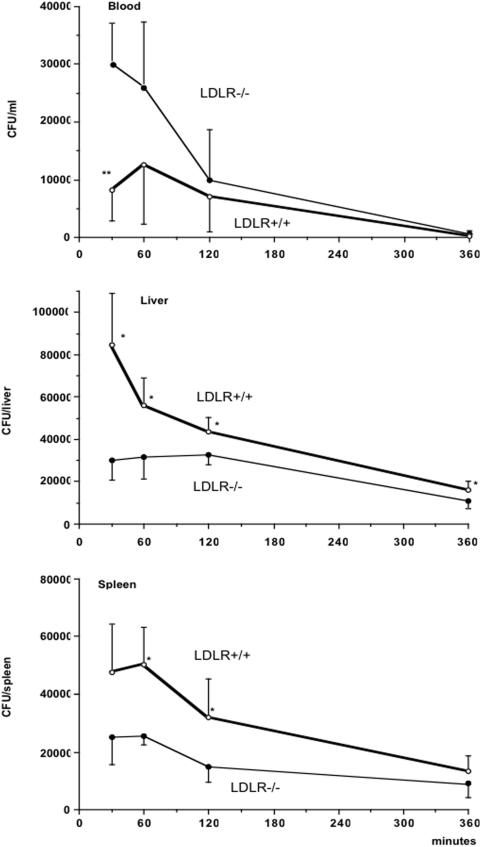
Hyperlipoproteinemia inhibits organ invasion by *S. typhimurium*. Distribution of *S. typhimurium* to the blood (cfu/ml), the liver (cfu/organ), and the spleen (cfu/organ) at various time points after i.v. injection of 10^5^ *Salmonella* cfu. Each point represents the mean±SD for at least 5 animals. Significant differences between LDLR−/− and LDLR+/+ mice are indicated (*p<0.05; **p<0.01; Mann-Whitney U test).

To investigate whether hyperlipoproteinemia influences the early organ invasion from the bloodstream by *Salmonella*, we determined the early distribution of the microorganisms after i.v. injection of 10^5^ *S. typhimurium* cfu. Blood from LDLR−/− mice contained significantly more *Salmonella* cfu than that of LDLR+/+ mice 30 minutes after injection (*P*<0.01), but an efficient elimination of the microorganisms occurred during the next 6 hours (Fig. 7A). The numbers of cfu in the liver and spleen were 70–80% lower in LDLR−/− mice than those in LDLR+/+ mice at 30 minutes after injection, and remained lower throughout the experiment, the difference between mouse strains being significant at 30, 60, 120 and 360 minutes for the liver (Fig. 7B, *P*<0.05) and at 30 and 60 minutes for the spleen (Fig. 7C, *P*<0.05).

## Discussion

In the present study, we demonstrate that hyperlipoproteinemic mice are resistant against *S. typhimurium* infection. The protection was not due to the absence of the LDLR in the knock-out mouse strain, but to a direct effect of hyperlipoproteinemia. The beneficial effect of lipoproteins was exerted by blocking the interaction of *Salmonella* with host cells, including endothelial cells and monocytes, which led to inhibition of organ invasion. This resulted in an altered distribution of the microorganism to the organs of the host, and increased survival.

It has previously been shown that lipoproteins bind and neutralize LPS, with beneficial effects in Gram-negative infections [5]–[8]. As *S. typhimurium* is an LPS-containing Gram-negative bacterium, and *Salmonella* LPS plays a crucial role in cytokine stimulation [17] and induction of mortality in vivo [18], an improved survival of LDLR−/− mice during systemic *S. typhimurium* infection would have been expected. Indeed, LDLR−/− mice were less susceptible to *S. typhimurium* infection, but this was not due to blunted cytokine production, as we observed undetectable or low cytokine circulating concentrations during the infection in the LDLR−/− and LDLR+/+ mice. In addition, the expression of cytokine mRNA was similar in the organs of LDLR−/− and LDLR+/+ mice. Thus, the cytokine response is not responsible for the mortality due to systemic *S. typhimurium* infection in this model, and the major difference is the almost complete absence of *Salmonella* in the organs of the LDLR−/− mice.

The low circulating cytokine response during systemic *S. typhimurium* infection may be attributed to the facultative intracellular nature of the organism. It should be noted that at later time points during the infection, the LDLR+/+ mice exhibited a greater cytokine response than did LDLR−/− mice, and this is likely due to the 1000-fold greater bacterial burden in the control mice, leading to substantial stimulation of the host response.

We hypothesized that the decreased bacterial growth in the LDLR−/− mice may be a result of enhanced phagocytosis and intracellular killing of *Salmonella* organisms in these mice. However, this proved not to be the case: phagocytosis and subsequent intracellular killing of the organisms by both neutrophils and macrophages did not differ between LDLR−/− and LDLR+/+ mice. Another reason for protection could have been the absence of the LDL receptor in the knock-out mice. *Toxoplasma gondii* is known to use the host LDLR for cholesterol acquisition [19], whereas penetration of cells by *Pseudomonas* exotoxin A is mediated through LDLR-related protein [20], [21]. Thus, use of the LDLR by *Salmonella* during organ invasion could be envisaged, but was ruled out by the observation that hyperlipoproteinemic ApoE−/− mice, which have an intact LDLR, also were resistant to *Salmonella* infection. In addition, LDL affected the adhesion of *S. typhimurium* to LDLR-bearing endothelial cells. This demonstrates that elevated lipoprotein concentrations, and not the lack of LDLR itself, are responsible from the resistance of mice against *Salmonella* infection.

Theoretically, there are several mechanisms that could account for the beneficial effects of the lipoproteins on *Salmonella* infection. Firstly, a direct effect of lipoproteins on the growth of *Salmonella* could be envisaged. In this respect, it is of interest that HDL has been found to be cidal against *Trypanosoma cruzi* [20]. However, the growth of *Salmonella* was similar in the plasma of LDLR−/−, ApoE−/− and control mice, excluding a direct antimicrobial effect of LDL. Secondly, lipoproteins may interact with *Salmonella*, putatively with its LPS component, and thus block bacterial binding and internalization by host cells. As LPS is crucial for the internalization of *Salmonella* [22], blocking the interaction between LPS and host cells may prevent subsequent tissue invasion. Indeed, preincubation of *Salmonella* with LDL led to reduced cytokine production, demonstrating that lipoproteins are able to inhibit the interaction of *Salmonella* with monocytes. Even more relevant for tissue invasion, preincubation of *Salmonella* with LDL significantly reduced its attachment to endothelial cells. This protective mechanism in which lipoproteins block *Salmonella* interaction with endothelial cells by their blockade of LPS represents the same type of mechanism as previously shown by the blockade of MSCRAMMs (microbial surface components recognizing adhesive matrix molecules) of *Staphylococcus* by naturally occurring antibodies, resulting in the inhibition of staphylococcal adhesion to endothelial cell and reduced tissue invasion [23].

Interaction of *Salmonella* with host cells likely is an important early step in the pathogenesis of invasive infection. The ability to infect tissue macrophages has been described as an invasive trait of intracellular bacteria such as *Salmonella* spp. [24], and attachment to endothelial cells is the first step in organ invasion by *Salmonella* from the bloodstream. The hypothesis that lipoproteins are able to directly modify organ invasion by *Salmonella*, was tested by assessing early distribution of *Salmonella* organisms in LDLR−/− and LDLR+/+ mice after i.v. injection of a large bacterial load. Indeed, we observed that invasion of *Salmonella* organisms into the liver and spleen of LDLR−/− mice was markedly lower than that in control mice. The difference in clearance of bacteria between the two strains of mice was already apparent within 30 minutes after injection. In this early phase of infection, the numerical balance between bacterial burden and host defense mechanisms in the tissues will determine the outcome of infection, and the LDLR−/− mice start with a substantial advantage over control animals. In contrast to the LDLR+/+ mice, the vast majority of *Salmonella* organisms in the LDLR−/− remain sequestrated in the circulation, where are eliminated by complement and neutrophils, known to efficiently clear *Salmonella* from the bloodstream [25]. Blocking tissue invasion by lipoproteins is not unique for *Salmonella*, as others have reported that VLDL inhibits liver invasion by *Plasmodium* sporozoites, leading to protection against malaria [26].

The precise molecular interaction between *Salmonella* and lipoproteins remains to be elucidated, but the bacterial LPS is the most likely candidate to be involved. *Salmonella* is known to interact with host cell Toll-like receptor (TLR)-4 through its LPS component [17], [27], and this signaling mechanism is likely blocked by binding of lipoproteins to the LPS. The exact nature of the lipoprotein particle responsible for interaction with *Salmonella* has yet to be identified. Phospholipids have been shown to mediate LPS neutralization, but protein components, such as apolipoprotein E, have also been reported to bind LPS [28]. Our findings in the apoE- and LDLR-deficient mice, which are also protected against salmonellosis, however, point to a binding site other than apoE. In addition to TLR4, the macrophage scavenger receptors [29], and the cystic fibrosis transmembrane conductance regulator protein [30] are probably involved in the entry of *Salmonella* into cells. Whether the interaction of these receptors with *Salmonella* is also influenced by lipoproteins remains to be elucidated.

In conclusion, plasma lipoproteins appear to be an important host defense mechanism against invasive *Salmonella* infection. A direct and rapid interaction between lipoproteins and *Salmonella* in the bloodstream occurs, preventing the invasion of the microorganisms from the bloodstream into the organs. These new insights improve the understanding of the pathogenesis of *Salmonella* infection, and could ultimately lead to the design of new therapeutic strategies.
